# A machine learning-based strategy to elucidate the identification of antibiotic resistance in bacteria

**DOI:** 10.3389/frabi.2024.1405296

**Published:** 2024-06-18

**Authors:** K. T. Shreya Parthasarathi, Kiran Bharat Gaikwad, Shruthy Rajesh, Shweta Rana, Akhilesh Pandey, Harpreet Singh, Jyoti Sharma

**Affiliations:** ^1^ Manipal Academy of Higher Education (MAHE), Manipal, Karnataka, India; ^2^ Institute of Bioinformatics, Bangalore, India; ^3^ Division of Biomedical Informatics, Indian Council of Medical Research, New Delhi, India; ^4^ Department of Laboratory Medicine and Pathology, Mayo Clinic, Rochester, MN, United States; ^5^ Center for Individualized Medicine, Mayo Clinic, Rochester, MN, United States

**Keywords:** pathogens, anti-microbial resistance, bioinformatics, machine learning, nucleotides, clustering

## Abstract

Microorganisms, crucial for environmental equilibrium, could be destructive, resulting in detrimental pathophysiology to the human host. Moreover, with the emergence of antibiotic resistance (ABR), the microbial communities pose the century’s largest public health challenges in terms of effective treatment strategies. Furthermore, given the large diversity and number of known bacterial strains, describing treatment choices for infected patients using experimental methodologies is time-consuming. An alternative technique, gaining popularity as sequencing prices fall and technology advances, is to use bacterial genotype rather than phenotype to determine ABR. Complementing machine learning into clinical practice provides a data-driven platform for categorization and interpretation of bacterial datasets. In the present study, k-mers were generated from nucleotide sequences of pathogenic bacteria resistant to antibiotics. Subsequently, they were clustered into groups of bacteria sharing similar genomic features using the Affinity propagation algorithm with a Silhouette coefficient of 0.82. Thereafter, a prediction model based on Random Forest algorithm was developed to explore the prediction capability of the k-mers. It yielded an overall specificity of 0.99 and a sensitivity of 0.98. Additionally, the genes and ABR drivers related to the k-mers were identified to explore their biological relevance. Furthermore, a multilayer perceptron model with a hamming loss of 0.05 was built to classify the bacterial strains into resistant and non-resistant strains against various antibiotics. Segregating pathogenic bacteria based on genomic similarities could be a valuable approach for assessing the severity of diseases caused by new bacterial strains. Utilization of this strategy could aid in enhancing our understanding of ABR patterns, paving the way for more informed and effective treatment options.

## 1 Introduction

Microorganisms/microbes are the oldest known life forms on Earth, dating back to approximately 3.42 billion years (Schopf et al., 2018). As the support system of the biosphere, these ubiquitous organisms are paramount for the survival of more complex organisms. They are involved in various intricate interactions including breakdown of biological components, food spoilage, climate change, and operation of basic metabolic cycles in plants (Omkar Khade, 2024). In addition to exercising these functions, several microorganisms have been reported as potential candidates for causing detrimental effects on other life forms. Such microbes that cause harm to the host form the class of pathogenic microorganisms. Salmonellosis, listeriosis, campylobacteriosis, yersiniosis, tuberculosis, gonorrhea, and syphilis are some of the life-threatening infections in humans caused by pathogenic microorganisms. In addition to the number of increasing infections by these microbes, another threat known as antibacterial resistance (ABR) has now taken a global turnover exhibiting the possibility of a future pandemic. A number of bacterial species have been identified as resistant to the available antibiotics that pose a threat to humanity in the near future (Ventola, 2015).

Several ecologists have come up with a broad spectrum of molecular techniques to investigate microbial communities (Davey and O’toole, 2000; Douterelo et al., 2014; Agrawal et al., 2015; Braga et al., 2016). These techniques aided not only in understanding the diversity among microbes but also in the characterization and selection of treatment strategies to overcome diseases caused by the pathogenic forms. With the extensive diversity and considerable number of known strains, characterization based on experimental techniques makes it expensive, labor-intensive, and time-consuming (Nemati et al., 2016; Qu et al., 2019). This reduces the potential for meta-analysis. Owing to the enormous amounts of data collected, microbiology has now emerged into a field with big data competencies (Falony et al., 2015; Kyrpides et al., 2016; Goodswen et al., 2021). Utilization of machine learning (ML) techniques for analysis of data has become a proven strategy in acquiring insights about microorganisms (Aida et al., 2022; Jiang et al., 2022; Munjal et al., 2022; Wu and Gadsden, 2023). Comprehensive studies on drug target prediction, drug resistance against antimicrobial drugs, prediction of disease outbreaks, and exploration of microbial–host interactions are now being carried out using ML techniques (Cazer et al., 2021; Kim and Ahn, 2021; Salim et al., 2021; Sudhakar et al., 2021; Kuang et al., 2022; Joshi et al., 2024). K-mer analysis and deep learning have been previously carried out to identify 16S short-read sequences from amplicon and shotgun data (Fiannaca et al., 2018). The tool MARVEL based on Random Forest algorithm aided in the prediction of dsDNA bacteriophage sequences from metagenomic studies (Amgarten et al., 2018).

Genetic programming, Random Forest, and logistic regression were previously used for the classification of microbes associated with bacterial vaginosis (Beck and Foster, 2014). Recently, another study showcased a new approach to analyze microbial–disease association through integration of multiple data sources (Fan et al., 2019). Similarly, a method for the diagnosis of malarial parasite(s) and for gastrointestinal parasite diagnosis was developed through binary image classification using convolutional neural network (Rajaraman et al., 2018; Mathison et al., 2020). Utilization of an support vector machine-based model for the prediction of secretory proteins from malarial parasites using amino acid compositions was another study that introduced ML in microbiology (Verma et al., 2008). Prediction of parasite load in the absence of quantitative polymerase chain reaction trained on clinical records of *Leishmania infantum*-infected dogs also indicated the application of ML in microbiology (Torrecilha et al., 2017). Certain studies have also investigated antimicrobial resistance (AMR) using ML-based approaches and have developed methods to classify genomes into resistant and susceptible against specific antibiotics (Drouin et al., 2016; Naidenov et al., 2019; Hyun et al., 2020). A study also presented the mapping of *Acinetobacter baumannii*, *Streptococcus pneumoniae*, *Staphylococcus aureus*, and *Mycobacterium tuberculosis* into three classes: susceptible, intermediate, and resistant (Davis et al., 2016). Similar studies by numerous research groups led to the development of methods for prediction of minimum inhibitory concentration (MIC) (Nguyen et al., 2018; Mujeeb et al., 2020; Umar et al., 2020; Valizadehaslani et al., 2020; Khan et al., 2021).

The current study employed ML-based algorithms and nucleotide sequences of pathogenic bacteria with humans as host and resistant to known antibiotics for clustering into groups of microorganisms sharing similar genomic features. Thereafter, a prediction model was developed to predict the cluster that is closest to the organism in question. Furthermore, the study includes the development of a multi-label classifier capable of predicting the antibiotic that the organism is resistant to, based on the cluster information. The clustering model was evaluated using the Silhouette coefficient, the Calinski–Harabasz index, and the Davies–Bouldin index (Caliński and Harabasz, 1974; David and Davies, 1979; Rousseeuw, 1987). The prediction models were evaluated on the basis of sensitivity, specificity, and 5-fold cross-validation (CV) accuracy. Although microbial infections involve the interplay of several molecular features, the pathogenic features corresponding to a certain pathogen remain unique to that pathogen (Voter et al., 2020; Liu et al., 2021; Parthasarathi et al., 2021). Here, the genes corresponding to the features selected in the prediction model were also identified that shed light on the biological importance of the features in distinguishing one strain from another. This study would aid in coming up with improved strategies for the segregation of pathogenic bacteria. Furthermore, based on genomic similarities and differences with other well-studied microorganisms, it may aid in assessing the severity of the disease produced by the bacterium. Furthermore, incorporating ML-based algorithms into clinical practice not only is viable, reproducible, and resilient, but also aids in the production of clinician-friendly outcomes. Overall, the computational prediction analyses directed the benefit of ML in clustering pathogenic bacterial forms, which may aid in the development of better strategies to improve treatment options.

## 2 Materials and methodology

**Figure 1:**
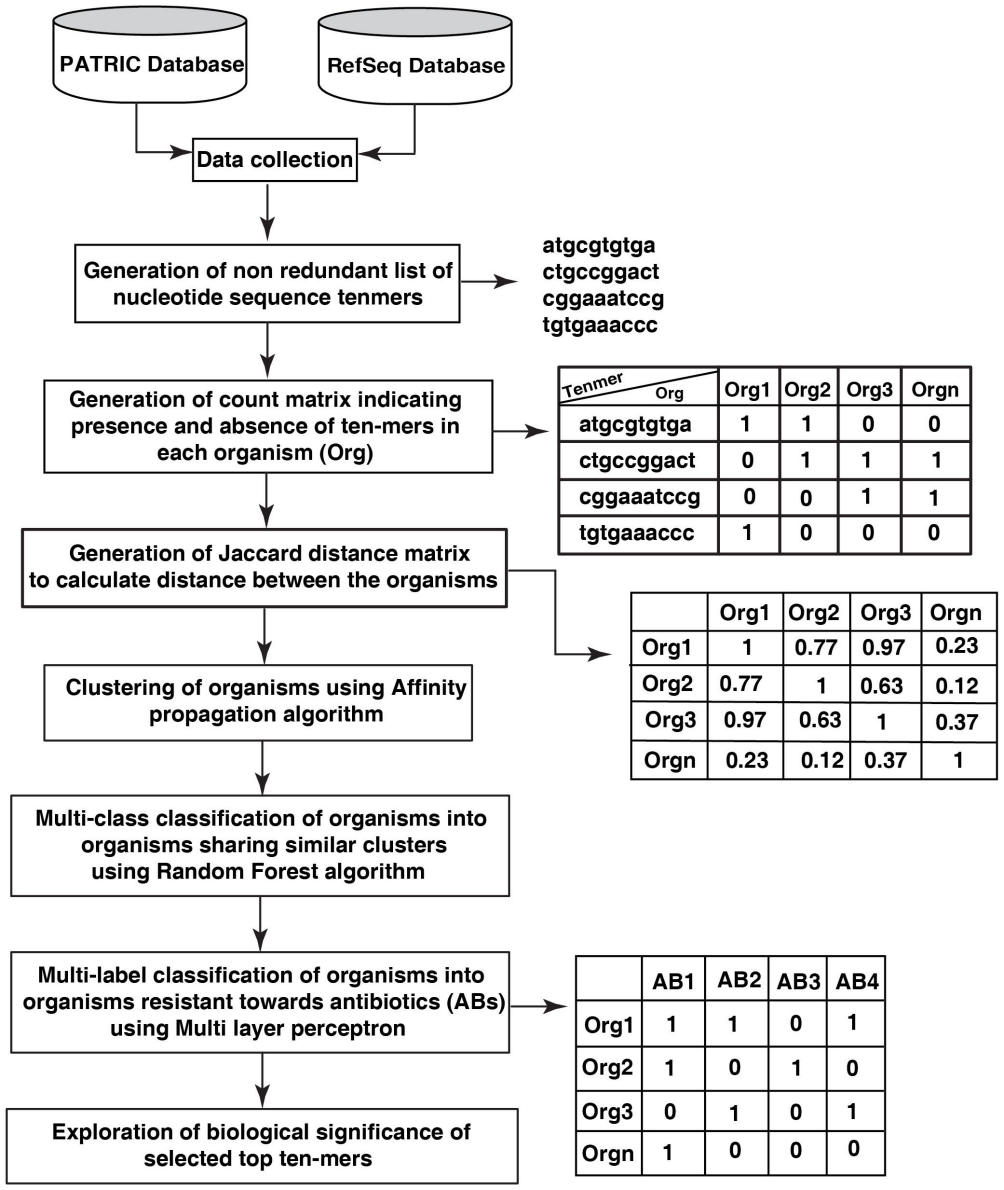
Depiction of workflow for building a prediction model for the identification of pathogenic organisms sharing similar characteristics.

The workflow of the study is depicted in **Figure 1**.

### 2.1 Data collection

A list of pathogenic bacteria resistant to antibiotics was obtained from the Pathosystems Resource Integration Center (PATRIC) database (Gillespie et al., 2011). The complete genomes for the microbes were downloaded from National Center for Biotechnology Information’s (NCBI) RefSeq database (Tatusova et al., 2016) (**Supplementary File**).

### 2.2 Generation of k-mer matrix from nucleotide sequences

Customized Python scripts were used to fragment the genomes into k-mers of length 8, 10, 12, and 14 nucleotides on a subset of bacterial sequences. The list of k-mers was filtered to remove duplicates. Thereafter, k-mers were mapped to the sequences, and a matrix containing the details on the presence and absence of the k-mers in each pathogenic strain was generated. R (v3.4.4) libraries seqinr and Biostrings were used to generate the matrix (Charif and Lobry, 2007; Pagès et al.). The clustering was performed on the subset for each k-mer length using the methodology as mentioned in *Section 2.3*. Thereafter, the optimum k-mer length for further processing was defined by taking into consideration the number of k-mers obtained, the amount of time required to process the k-mers, the intermediate file sizes, and the goodness of clustering.

### 2.3 Segregation of pathogenic microbes

The distance between the species was calculated using Jaccard distance matrices. Thereafter, the values in the distance matrix were scaled and used as input to perform principal component analysis (PCA) (Prokopenko et al., 2016). Principal component 1 (PC1) was used as input to segregate the organism strains into different clusters using the unsupervised machine learning algorithm—Affinity propagation (Frey and Dueck, 2007). This allowed clustering of organism strains based on their genomic sequence similarities. Thereafter, the Silhouette coefficient, the Calinski–Harabasz index, and the Davies–Bouldin index were used to calculate the goodness of clustering obtained. The Silhouette coefficient ranges from −1 to +1 and a value close to +1 indicates a better-defined cluster. Higher values of the Calinski–Harabasz index indicate better separation between clusters while a lower Davies–Bouldin value corresponds to better separation between clusters. The scikit-learn library from Python v3.10 was used to segregate the organisms into different clusters (Fabian Pedregosa). Seaborn and matplotlib libraries were used for graphical visualizations (Hunter, 2007; Waskom, 2021).

### 2.4 Development of prediction model to predict the cluster of an organism

The clusters formed using the Affinity propagation algorithm were further used as class labels in supervised Random Forest algorithm to develop a prediction model. The binary matrix with the information on the presence and absence of k-mers along with the clusters was given as input to the Random Forest algorithm. The dataset was split into a train and test set in the ratio 80:20. Feature selection was performed using the Random Forest algorithm, and the k-mers (features) with a score > 0.0001 were selected as most informative k-mers. The train set was further divided into a train and a validation set and the hyperparameters were tuned on the validation set using Python v3.10 library GridSearchCV (Lavalle et al., 2004). The model was evaluated based on sensitivity and specificity. The model was built using customized scripts written in Python v3.10 using the scikit-learn library. The model was then saved using the joblib library (Varoquaux, 2023).

### 2.5 Determining the biological significance of the most informative k-mers in cluster prediction

The k-mers selected in the Random Forest model were further analyzed to determine their biological significance. Standalone Basic Local Alignment Search Tool (BLAST) (v2.15.0) was used to align the k-mers with the customized database generated using the gene sequences from various reference genomes of bacterial strains downloaded from the Database of Essential Genes (DEG) (Luo et al., 2021) (last update, 2020 September 1) (Camacho et al., 2009). The alignments with 100% identity using the “blastn-short” parameter of Standalone BLAST were saved (Camacho et al., 2009). The list of genes obtained after alignment was compared with the list of known AMR genes obtained from the Comprehensive Antibiotic Resistance Database (CARD) (Alcock et al., 2023).

### 2.6 Development of prediction model of microbes resistant towards antibiotics

Furthermore, the data collected from the PATRIC database on resistance of a strain towards different antibiotics were used along with the clustering output to develop a multi-label prediction model for predicting the putative antibiotics that may not be useful in treatment against a specific bacterial strain. Given a nucleotide sequence, the model calculated several genomic features including GC content and mononucleotide counts of each of the strains. This information was merged with the clustering output, and a binary matrix with details on whether an organism strain is resistant to a specific antibiotic was generated. A multi-label classification model was built assuming that resistance against each antibiotic was independent of the fact that a strain is resistant to the other antibiotics. A multi-layer perceptron (MLP) with the calculated features representing the input layers, multiple hidden layers, and an output layer representing the resistance to antibiotics was generated. The Rectified Linear Unit (ReLU) activation function was applied for the hidden layers and the binary cross-entropy loss and the Adam version of stochastic gradient descent method was implemented for weight updation (Fukushima, 1975; Diederik and Kingma, 2017). The sigmoid activation function was implemented for the output layers. The model was built using customized scripts written in Python v3.10 utilizing the keras and scikit-learn libraries (Chollet, 2015). 5-fold CV with hamming loss as the accuracy measure was used to grade the performance of the prediction model. Hamming loss evaluates individual label prediction rather than label combination. A lower hamming loss would thus indicate a better model.

## 3 Results

### 3.1 Collection of data

A list of 710 strains from seven genera of pathogenic bacterial strains resistant to 63 antibiotics was obtained from the PATRIC database. The bacterial complete genome sequences were downloaded from the NCBI RefSeq database. **Table 1** summarizes the number of strains included in the present study.

**Table 1:** Total number of strains from different genera included in the study.

Total	*Acinetobacter*	*Escherichia*	*Mycobacterium*	*Pseudomonas*	*Salmonella*	*Staphylococcus*	*Streptococcus*
710	39	42	99	137	28	305	60

### 3.2 k-mer matrix generation

The k-mers of length 10 nucleotides (10-mers) were selected as the optimum size of k-mers (**Supplementary File**). A total of 2,136,154 10-mers were obtained from the 710 strains. Filtering out redundant and 39,032 10-mers that contained nucleotides other than adenine, guanine, thymine, and cytosine yielded 1,048,573 unique 10-mers. A binary matrix with the dimensions 710:1,048,573 was obtained, consisting of rows representing strains, columns representing 10-mers, and cells with binary values signifying the presence or absence of the 10-mer in the individual strain.

### 3.3 Clustering pathogenic bacteria

**Figure 2:**
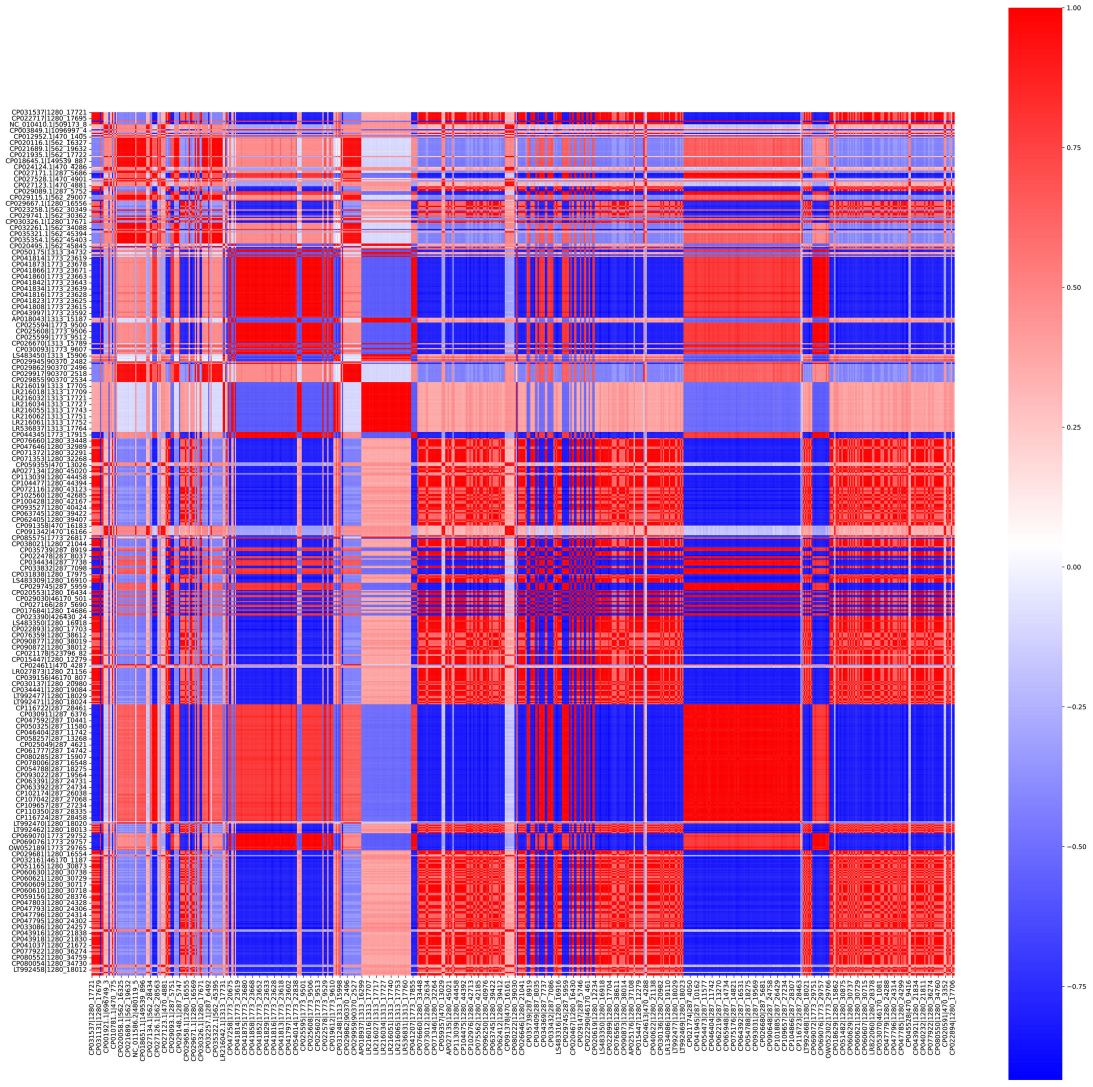
Heatmap representing the distance between the different bacterial strains calculated using Jaccard distance matrix with binary matrix of 10-mers as input.

**Figure 3:**
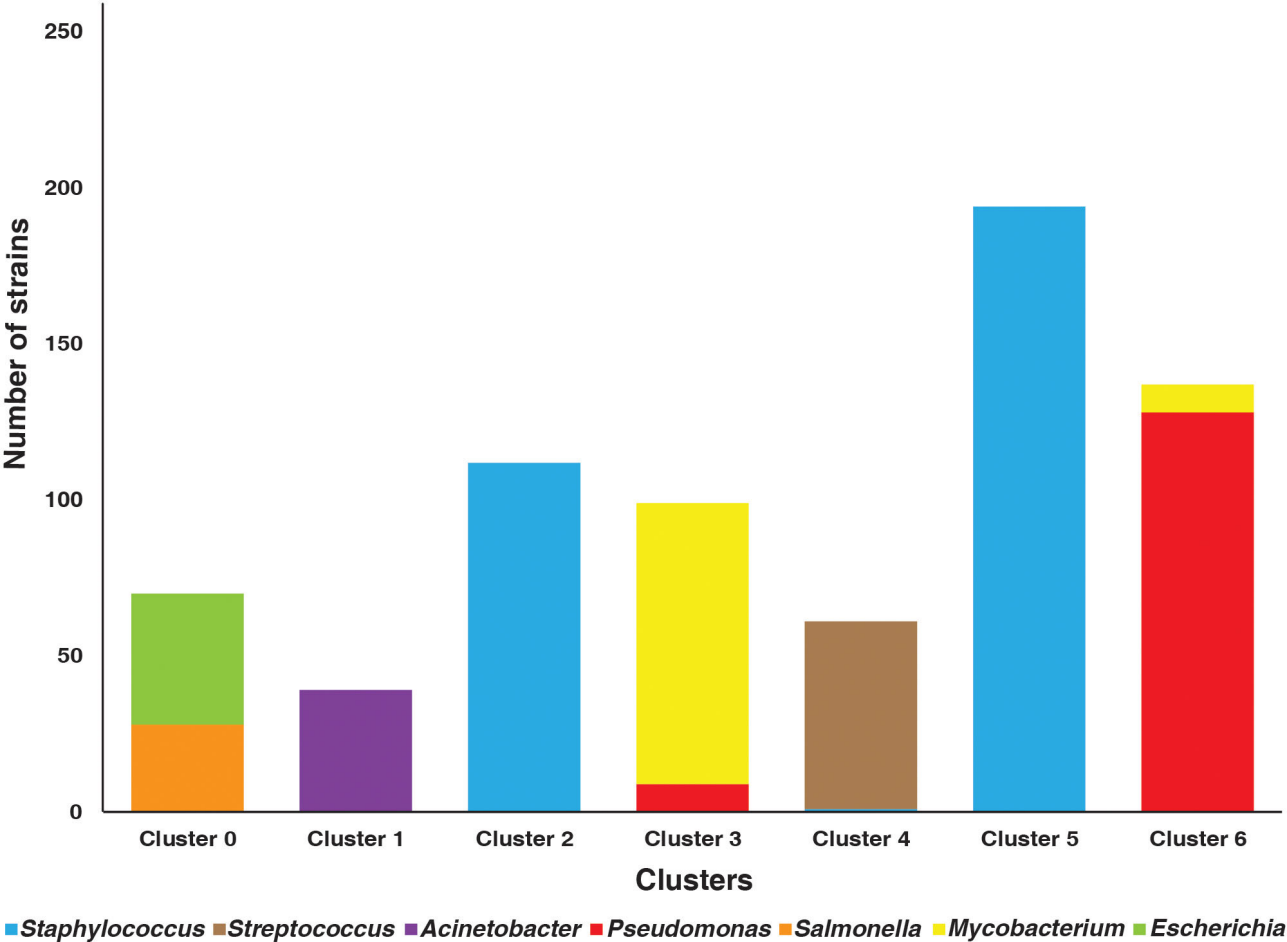
Bar plot representing the number of strains clustered in strains from various genera.

The distance between the pathogenic bacterial strains on the basis of the presence and absence of 10-mers was calculated using Jaccard distance matrices (**Figure 2**). The bacterial strains were clustered into seven clusters. A Silhouette coefficient of 0.82, a Calinski–Harabasz index of 93,672.21, and a Davies–Bouldin index of 0.19 were obtained (**Supplementary Table 1**; **Figure 3**).

### 3.4 Supervised ML model from the clusters obtained

The dataset with information on the presence and absence of 10-mers as features and the clusters as class labels was split into a train and a test set in the ratio 80:20. This accounted for 568 data points in the train set and 142 data points in the test set. Thereafter, the train set was further split into a train and a validation set in the ratio 80:20, resulting in 454 data points in the train set and 114 data points in the validation set. A total of 876 10-mers were selected as the most informative 10-mers using the Random Forest algorithm for feature selection. A Random Forest model with a maximum depth of 6, minimum samples leaves set to 2, minimum samples split set to 5, number of estimators set to 100, and criterion set as Gini was generated. 5-fold CV on the validation set using the set parameters resulted in an accuracy of 96.49%. Testing the model on the test set resulted in an overall sensitivity of 0.98 and a specificity of 0.99. **Table 2** mentions the individual class sensitivity and specificity.

**Table 2:** Sensitivity and specificity of individual cluster prediction.

Cluster	Sensitivity	Specificity
0	1.00	1.00
1	1.00	1.00
2	1.00	1.00
3	0.99	0.95
4	1.00	1.00
5	1.00	1.00
6	0.99	0.96

### 3.5 Biological significance of 876 10-mers

All the 876 10-mers mapped to 26,058 entries from DEG that corresponded to 81 bacterial strains, 5,179 unique genes, and 8,752 unique proteins including putative and hypothetical proteins (**Supplementary Table 2**). **Table 3** summarizes the number of 10-mers mapped to the strains from species included in the current study.

**Table 3:** Number of 10-mers mapped to reference species included in the current study.

Species	No. of 10-mers	Genes	Proteins
*Acinetobacter baumannii*	834	120	491
*Escherichia coli*	876	1,092	1,841
*Mycobacterium tuberculosis*	801	983	1,119
*Pseudomonas aeruginosa*	788	652	765
*Salmonella enterica*	854	525	606
*Staphylococcus aureus*	816	816	649
*Streptococcus pneumoniae*	673	241	194

**Figure 4:**
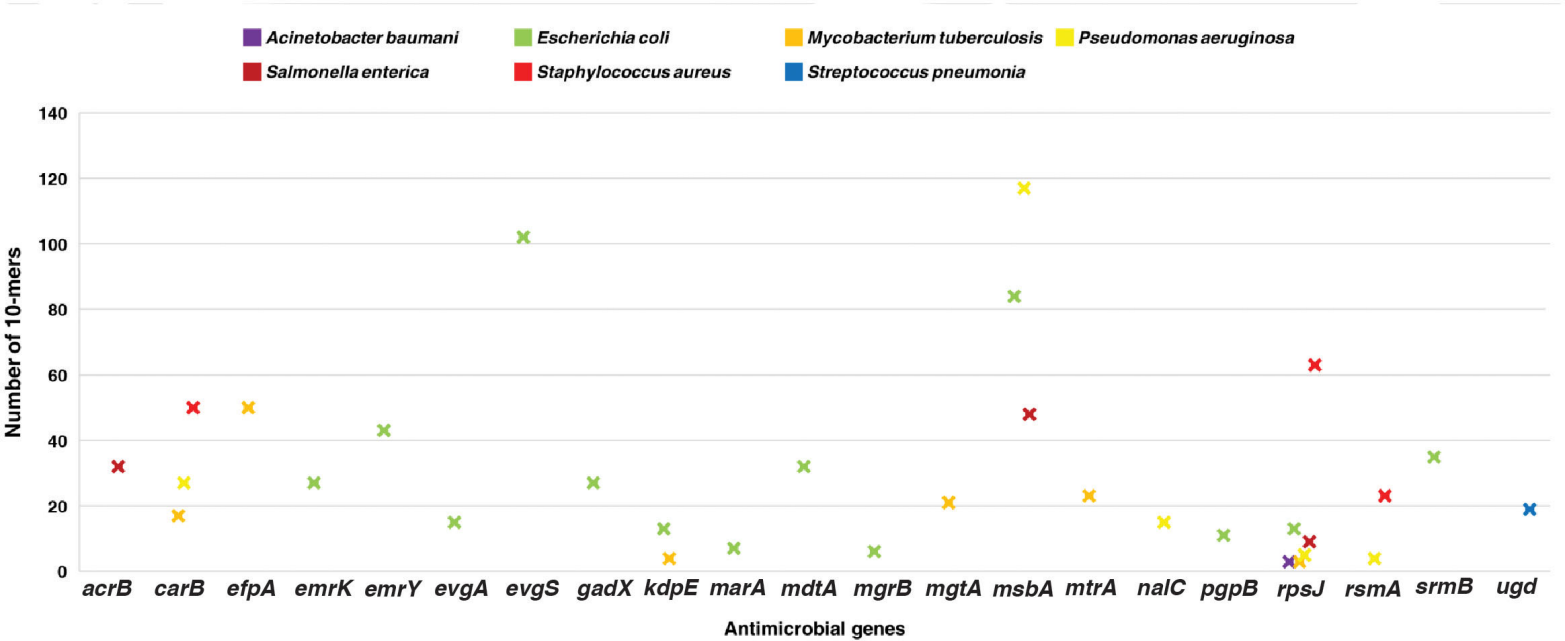
Scatter plot indicating the number of 10-mers mapped to ABR drivers in strains of different genera.

A total of 703 10-mers mapped to 30 genes known to cause AMR from CARD. Of those, 448 10-mers belonged to the organisms considered in the current study and mapped to 22 AMR genes. The 10-mers corresponding to *Escherichia coli* mapped to 13 AMR-related genes, namely, *emrK*, *emrY*, *evgA*, *evgS*, *gadX*, *kdpE*, *marA*, *mdtA*, *mgrB*, *msbA*, *pgpB*, *rpsJ*, and *srmB*. Certain 10-mers mapped to AMR-related genes in only one species, namely, *efpA*, *mgtA*, and *mtrA* mapped exclusively to *M. tuberculosis*-related 10-mers, and *acrB* mapped exclusively to *Salmonella enterica*-related 10-mers. Similarly, there were AMR-related genes that mapped exclusively to *E.coli*, *Pseudomonas aeruginosa*-, and *S. pneumoniae*-related 10-mers (**Supplementary Table 3**). **Figure 4** summarizes the number of 10-mers mapped to individual AMR genes in specific species.

### 3.6 Prediction model for identifying putative antibiotic resistance in bacterial strain

**Figure 5:**
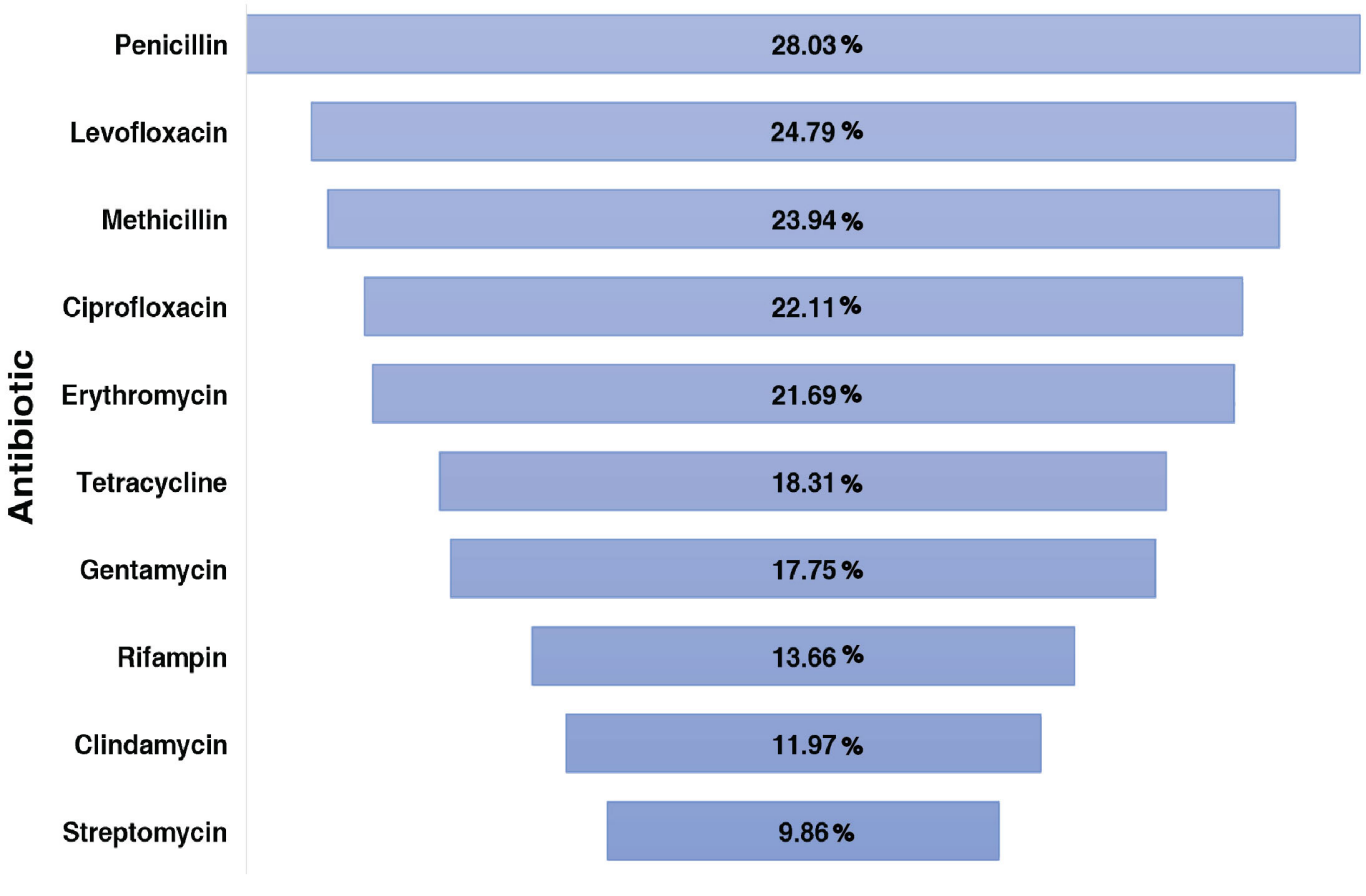
Funnel plot representing the top 10 antibiotics identified to be resistant by the bacterial strains.

The 710 bacterial strains used in the study were resistant towards 63 different antibiotics. **Figure 5** summarizes the top 10 antibiotics found most commonly resistant among different strains in the present study. **Supplementary Table 4** summarizes the list of bacterial strains resistant to the 63 antibiotics.

The length of the different strains ranged from 391,326 base pairs (bps) to 7,267,567 bps with an average length of 4,017,469 bps. The strains belonging to *P. aeruginosa* species had the largest genomes along with a high GC content. However, the strains belonging to *E. coli* had high “A” and “T” mononucleotide counts (**Supplementary Table 5**). The MLP model with 20 hidden layers was developed. The hamming loss calculated using repeated 5-fold CV was 0.05, indicating the prediction to be false 5% of times.

## 4 Discussion

Advances in the processing capacity, improvements in the classical data processing algorithms, and the availability of bacterial whole genome sequences (WGS) in public databases allow for a retrospective population study of many bacterial populations. Identifying patterns in the genomic sequences resulting in mosaic structures poses challenges in comprehending and visualizing the diversity and similarities within and across various bacterial strains. However, increasing interest in the quantitative techniques to predict phenotypes from genotypes beginning with bacterial WGS are becoming popular. The pathogenicity and ABR could be the key phenotypes for predicting clinical outcomes and estimating possible treatment options.

The present study emphasizes on the utilization of ML-based techniques to examine the relatedness in the different bacterial strains. Clustering analysis was performed to segregate the pathogenic forms based on their genomic similarities. Various species undergo horizontal gene transfer in the evolution process to increase their chances of survival (Burmeister, 2015). The most evident advantage of horizontal gene transfer is that a cell can acquire a beneficial gene that originated in another cell. The emergence of new beneficial genes is likely extremely rare; therefore, stealing a gene from a neighbor should be considerably faster than waiting for it to evolve independently (Vogan and Higgs, 2011). Moreover, it would also allow a cell to reclaim a gene that had been lost by another member of the population (Vogan and Higgs, 2011). Horizontal gene transfer can also acquire beneficial features that aid adaptation to new environments, such as metabolic and antibiotic resistance genes (Hall et al., 2020). This enables organisms to become interdependent, ensuring cooperation in preserving their relationship (Hall et al., 2020). This phenomenon could be visualized in the present study as the strains from *Escherichia*, *Salmonella*, *Mycobacterium*, and *Pseudomonas* did not form single clusters. The strains belonging to *E. coli* and *S. enterica* clustered together, indicating the strains within these species to share similarities. Among the 10-mers selected, 854 10-mers mapped to *Salmonella* strains while 876 k-mers mapped to *Escherichia* strains. All the 10-mers belonging to *Salmonella* overlapped with the k-mers from *Escherichia.* A total of 342 genes identified based on the selected 10-mers were common among the two organisms (**Supplementary Table 2**). The two species are a part of the same family—Enterobacteriaceae. According to evolutionary rate estimates derived from 5S and 16S rRNA sequence analysis, *Escherichia* and *Salmonella* species diverged from a common ancestor (Bisi-Johnson et al., 2011). They are estimated to have separated from the common ancestor approximately 140 million years ago (Ochman and Wilson, 1987; Hu et al., 2010). Despite their contrasting lifestyles, there has been no significant rewiring at the level of local regulons involved (Peyman Zarrineh et al., 2014). There is notable conservation in signaling pathways and stress sensing across these phylogenetically similar species (Peyman Zarrineh et al., 2014). Moreover, a similarity of 76% to 100% between their housekeeping genes makes them evolutionarily closely related species (Sharp, 1991; Samuel et al., 2004; Hu et al., 2010). They are foodborne pathogens and create complex biofilms that contribute to their virulence, antibiotic resistance, and surface survival (Milho et al., 2019). Interspecies interactions occur in mixed biofilms, resulting in diverse consequences for each species (Milho et al., 2019).

Furthermore, two clusters, cluster 3 and cluster 6, had a mixture of *Mycobacterium* and *Pseudomonas* pathogenic strains. These two clusters comprised a small fraction of strains joining the opponent cluster. Species collaborate when they are mutually advantageous, when their interests are aligned, and when each individual improves the fitness of the other, thus encouraging the advancement of diverse, unique phenotypes or interactions (Hall et al., 2020). One of the most common forms of prokaryote cooperation is the secretion of products required to build biofilms, digest complex chemicals, and modulate a host’s immune response, among other important functions (Hall et al., 2020). Genes involved in such benefit production can be transmitted between organisms, opening up new possibilities for collaboration and adaptation (Hall et al., 2020).

Currently, there are no studies indicating an evolutionary relationship between *Mycobacterium* and *Pseudomonas* bacteria. However, there is evidence of the two pathogens interacting to co-colonize the same infection niches and create a mixed-species biofilm that enhances both their immune system and antibiotic resistance (Camus et al., 2022). Further studies are needed to understand their evolutionary and clinical phenotype implications. Another interesting finding from this study was that strains belonging to *S. aureus* segregated to two separate clusters, indicating the within-species diversity. In the course of evolution, it undergoes both horizontal and vertical gene transfer events that have resulted in the genetically diversified bacterial population (Furqan Awan et al., 2021). Their diversity makes them resistant towards almost all the antimicrobial drugs used (Mlynarczyk-Bonikowska et al., 2022).

The obtained clusters paved the way for the introduction of a strategy based on the Random Forest algorithm to segregate the strains into organisms sharing similar genomic features. The proposed model attempts to integrate genomic sequences of the disease-causing microbes and further cluster them into groups of pathogens sharing similar characteristics. Along with developing the model, the study also identified key 10-mers that were capable of differentiating the strains into clusters. This could significantly accelerate the processing time required to deliver the output in terms of cluster identification. The majority of 10-mers mapping to genes in each organism varied across organisms.

Of the 876 10-mers, majority of them mapped to *gltB* gene in *Acinetobacter baumannii*. It codes for glutamate synthase subunit alpha. Glutamate is one of the carbon sources that can support growth of *Acinetobacter* species, making glutamate synthetases an important protein in these organisms (Ren and Palmer, 2023).

Majority of 10-mers corresponding to *E. coli* mapped to *toxB* gene. *toxB* is a virulence gene present in the virulence plasmid of *E. coli* species. It functions in enhancing bacterial adhesion and in inhibiting host lymphocyte activation (Tozzoli et al., 2005).

The *fas* gene stood out within 10-mers in *M. tuberculosis* strains. Biosynthesis of fatty acids regulated by FAS-I polypeptide is crucial in the formation of mycobacterial cell wall components, specifically mycolic acids that form a protective lipid layer on the cell wall. This is required for the survival of the bacterium in the host environment (Kinsella et al., 2003; Apoorva Bhatt et al., 2007). *rpoB* gene was the most common gene in the 10-mers mapped to *P. aeruginosa* and *S. aureus*. The list of 10-mers mapped were different in both the species, although there were some overlaps. These two species form one of the most commonly observed clinical polymicrobial communities that lead to the emergence of antibiotic-resistant strains (Pajon et al., 2023). *rpoB* gene is a DNA-directed RNA polymerase, and studies have reported that mutations in this gene lead to resistance against rifampin, an antibiotic used against multidrug-resistant bacterial strains (Yee et al., 1996; Guo et al., 2021). In *S. enterica*-related 10-mers, majority of them mapped to *ftsK*. It is involved in cell division and peptidoglycan biosynthesis. Mutations in *ftsK* could result in increased susceptibility against β-lactams and ciprofloxacin-related tolerance (Curiao et al., 2016). The gene *spr0328* was identified as the topmost gene in *S. pneumoniae*. It encodes for a conserved hypothetical protein with a role in cell wall surface anchorage. The protein was one of the selected candidates for a study related to vaccine testing due to its ability to raise immune response in infected patients (Olaya-Abril et al., 2013).

The mapping of 10-mers to ABR genes led to the exploration of developing an MLP-based model to predict the antibiotics that a specific strain could be resistant to due to its genomic properties. This could thus aid in tracking ABR strains in a time-efficient manner. The 10-mers identified in the present study could open up new avenues in the field of drug designing-based studies. However, the present study is based on a limited number of sequences, although the same model could be implemented to a larger bacterial cohort based on sequence availability.

Amid growing advances in whole genome sequencing and applications of ML-based techniques, the characterization of pathogenic microbial communities could become a rapid process in the near future. The current study demonstrates one such strategy in identifying bacterial strains based on the presence and absence of 10-mers in their genomes. A subset of 10-mer sequences across the strains in the present study could also act as signatures to explore the diversity through understanding their biological significance. Furthermore, the MLP model enabled the classification of strains to ABR and non-ABR strains against various antibiotics. Overall, the computational prediction analyses demonstrated the advantage of ML to uncover the ABR determinants that might facilitate the exploration of better treatment options. However, the study is a data-driven approach, and thus, outcomes of the study may appear in the form of overfitting or underfitting.

## Data availability statement

The original contributions presented in the study are included in the article/**Supplementary Material**. Further inquiries can be directed to the corresponding author. Customized Python scripts used for developing the model are available through the GitHub repository via the following URL: https://github.com/js-iob/Bacterial_clustering_AMR.

## Author contributions

KTSP: Data curation, Formal analysis, Investigation, Methodology, Resources, Software, Validation, Visualization, Writing – original draft, Writing – review & editing. KG: Data curation, Methodology, Resources, Software, Writing – review & editing. SRaj: Data curation, Methodology, Writing – review & editing. SRan: Writing – review & editing. AP: Funding acquisition, Supervision, Writing – review & editing. HS: Writing – review & editing. JS: Conceptualization, Funding acquisition, Investigation, Methodology, Supervision, Writing – review & editing.

## References

[B1] Agrawal P. K. Agrawal S. Shrivastava R. (2015). Modern molecular approaches for analyzing microbial diversity from mushroom compost ecosystem. 3 Biotech. 5, 853–866. doi: 10.1007/s13205-015-0289-2 PMC4624149 28324393

[B2] Aida H. Hashizume T. Ashino K. Ying B. W. (2022). Machine learning-assisted discovery of growth decision elements by relating bacterial population dynamics to environmental diversity. Elife 11. doi: 10.7554/eLife.76846.sa2 PMC9417415 36017903

[B3] Alcock B. P. Huynh W. Chalil R. Smith K. W. Raphenya A. R. Wlodarski M. A.. (2023). CARD 2023: expanded curation, support for machine learning, and resistome prediction at the Comprehensive Antibiotic Resistance Database. Nucleic Acids Res. 51, D690–D699. doi: 10.1093/nar/gkac920 36263822 PMC9825576

[B4] Amgarten D. Braga L. P. P. Da Silva A. M. Setubal J. C. (2018). MARVEL, a tool for prediction of bacteriophage sequences in metagenomic bins. Front. Genet. 9, 304. doi: 10.3389/fgene.2018.00304 30131825 PMC6090037

[B5] Apoorva Bhatt V. M. Gurdyal S. Besra W. R., Laurent Kremer J. Jr (2007). The Mycobacterium tuberculosis FAS-II condensing enzymes: their role in mycolic acid biosynthesis, acid-fastness, pathogenesis and in future drug development. Mol. Microbiol. 64, 1442–1454. doi: 10.1111/j.1365-2958.2007.05761.x 17555433

[B6] Beck D. Foster J. A. (2014). Machine learning techniques accurately classify microbial communities by bacterial vaginosis characteristics. PloS One 9, e87830. doi: 10.1371/journal.pone.0087830 24498380 PMC3912131

[B7] Bisi-Johnson M. A. Obi C. L. Vasaikar S. D. Baba K. A. Hattori T.. (2011). Molecular basis of virulence in clinical isolates of Escherichia coli and Salmonella species from a tertiary hospital in the Eastern Cape, South Africa. Gut Pathog. 3. doi: 10.1186/1757-4749-3-9 PMC3125331 21663681

[B8] Braga R. M. Dourado M. N. Araujo W. L. (2016). Microbial interactions: ecology in a molecular perspective. Braz. J. Microbiol. 47 Suppl 1, 86–98. doi: 10.1016/j.bjm.2016.10.005 27825606 PMC5156507

[B9] Burmeister A. R. (2015). Horizontal gene transfer. Evol. Med. Public Health 2015, 193–194. doi: 10.1093/emph/eov018 26224621 PMC4536854

[B10] Caliński T. Harabasz J. (1974). A dendrite method for cluster analysis. Commuications Stat 3, 1–27. doi: 10.1080/03610927408827101

[B11] Camacho C. Coulouris G. Avagyan V. Ma N. Papadopoulos J. Bealer K.. (2009). BLAST+: architecture and applications. BMC Bioinf. 10, 421. doi: 10.1186/1471-2105-10-421 PMC2803857 20003500

[B12] Camus L. Briaud P. Vandenesch F. Doleans-Jordheim A. Moreau K. (2022). Mixed Populations and Co-Infection: Pseudomonas aeruginosa and Staphylococcus aureus. Adv. Exp. Med. Biol. 1386, 397–424. doi: 10.1007/978-3-031-08491-1_15 36258081

[B13] Cazer C. L. Westblade L. F. Simon M. S. Magleby R. Castanheira M. Booth J. G.. (2021). Analysis of multidrug resistance in staphylococcus aureus with a machine learning-generated antibiogram. Antimicrob. Agents Chemother. 65. doi: 10.1128/AAC.02132-20 PMC8097487 33431415

[B14] Charif D. Lobry J. R. (2007). SeqinR 1.0–2: A Contributed Package to the R Project for Statistical Computing Devoted to Biological Sequences Retrieval and Analysis. Springer, Berlin, Heidelberg. doi: 10.1007/978-3-540-35306-5_10

[B15] Chollet F. (2015). Keras (GitHub repository: GitHub). Available at: https://github.com/fchollet/keras.

[B16] Curiao T. Marchi E. Grandgirard D. Leon-Sampedro R. Viti C. Leib S. L.. (2016). Multiple adaptive routes of Salmonella enterica Typhimurium to biocide and antibiotic exposure. BMC Genomics 17, 491. doi: 10.1186/s12864-016-2778-z 27411385 PMC4943003

[B17] Davey M. E. O’toole G. A. (2000). Microbial biofilms: from ecology to molecular genetics. Microbiol. Mol. Biol. Rev. 64, 847–867. doi: 10.1128/MMBR.64.4.847-867.2000 11104821 PMC99016

[B18] David L. Davies D. W. B. (1979). A cluster separation measure. IEEE Trans. Pattern Anal. Mach. Intelligence. 224–227. doi: 10.1109/TPAMI.1979.4766909 21868852

[B19] Davis J. J. Boisvert S. Brettin T. Kenyon R. W. Mao C. Olson R.. (2016). Antimicrobial resistance prediction in PATRIC and RAST. Sci. Rep. 6, 27930. doi: 10.1038/srep27930 27297683 PMC4906388

[B20] Diederik P. Kingma J. L. B. (2017). ADAM: A METHOD FOR STOCHASTIC OPTIMIZATION. ICLR 2015. doi: 10.48550/arXiv.1412.6980

[B21] Douterelo I. Boxall J. B. Deines P. Sekar R. Fish K. E. Biggs C. A. (2014). Methodological approaches for studying the microbial ecology of drinking water distribution systems. Water Res. 65, 134–156. doi: 10.1016/j.watres.2014.07.008 25105587

[B22] Drouin A. Giguere S. Deraspe M. Marchand M. Tyers M. Loo V. G.. (2016). Predictive computational phenotyping and biomarker discovery using reference-free genome comparisons. BMC Genomics 17, 754. doi: 10.1186/s12864-016-2889-6 27671088 PMC5037627

[B23] Fabian Pedregosa G. V. Gramfort A. Michel V. Thirion B. Grisel O. Blondel M.. Scikit-learn: Machine Learning in Python. Available online at: https://www.jmlr.org/papers/v12/pedregosa11a.html.

[B24] Falony G. Vieira-Silva S. Raes J. (2015). Microbiology Meets big data: the case of gut microbiota-derived trimethylamine. Annu. Rev. Microbiol. 69, 305–321. doi: 10.1146/annurev-micro-091014-104422 26274026

[B25] Fan C. Xiujan L. Guo L. Zhang A. (2019). Predicting the associations between microbes and diseases by integrating multiple data sources and path-based HeteSim scores. Neurocomputing 323, 76–85. doi: 10.1016/j.neucom.2018.09.054

[B26] Fiannaca A. La Paglia L. La Rosa M. Lo Bosco G. Renda G. Rizzo R.. (2018). Deep learning models for bacteria taxonomic classification of metagenomic data. BMC Bioinf. 19, 198. doi: 10.1186/s12859-018-2182-6 PMC6069770 30066629

[B27] Frey B. J. Dueck D. (2007). Clustering by passing messages between data points. Science 315, 972–976. doi: 10.1126/science.1136800 17218491

[B28] Fukushima K. (1975). Cognitron: A self-organizing multilayered neural network. Biol. Cybernetics. 20, 121–136. doi: 10.1007/BF00342633 1203338

[B29] Furqan Awan M. M. A. Hassan Mushtaq M. Ijaz M. (2021). Genetic Diversity in Staphylococcus aureus and Its Relation to Biofilm Production. Intechopen.

[B30] Gillespie J. J. Wattam A. R. Cammer S. A. Gabbard J. L. Shukla M. P. Dalay O.. (2011). PATRIC: the comprehensive bacterial bioinformatics resource with a focus on human pathogenic species. Infect. Immun. 79, 4286–4298. doi: 10.1128/IAI.00207-11 21896772 PMC3257917

[B31] Goodswen S. J. Barratt J. L. N. Kennedy P. J. Kaufer A. Calarco L. Ellis J. T. (2021). Machine learning and applications in microbiology. FEMS Microbiol. Rev. 45. doi: 10.1093/femsre/fuab015 PMC8498514 33724378

[B32] Guo Y. Rao L. Wang X. Zhao H. Li M. Yu F. (2021). Molecular characteristics of rifampin-sensitive and -resistant isolates and characteristics of rpoB gene mutations in methicillin-resistant staphylococcus aureus. Dovepress. 14, 4591–4600. doi: 10.2147/IDR.S336200 PMC8576291 34764656

[B33] Hall R. J. Whelan F. J. Mcinerney J. O. Ou Y. Domingo-Sananes M. R. (2020). Horizontal gene transfer as a source of conflict and cooperation in prokaryotes. Front. Microbiol. 11, 1569. doi: 10.3389/fmicb.2020.01569 32849327 PMC7396663

[B34] Hu B. Perepelov A. V. Liu B. Shevelev S. D. Guo D. Senchenkova S. N.. (2010). Structural and genetic evidence for the close relationship between Escherichia coli O71 and Salmonella enterica O28 O-antigens. FEMS Immunol. Med. Microbiol. 59, 161–169. doi: 10.1111/j.1574-695X.2010.00676.x 20482625

[B35] Hunter J. D. (2007). Matplotlib: A 2D graphics environment. Computing Sci. Eng. 9, 90–95. doi: 10.1109/MCSE.2007.55

[B36] Hyun J. C. Kavvas E. S. Monk J. M. Palsson B. O. (2020). Machine learning with random subspace ensembles identifies antimicrobial resistance determinants from pan-genomes of three pathogens. PloS Comput. Biol. 16, e1007608. doi: 10.1371/journal.pcbi.1007608 32119670 PMC7067475

[B37] Jiang Y. Luo J. Huang D. Liu Y. Li D. D. (2022). Machine learning advances in microbiology: A review of methods and applications. Front. Microbiol. 13, 925454. doi: 10.3389/fmicb.2022.925454 35711777 PMC9196628

[B38] Joshi G. Jain A. Araveeti S. R. Adhikari S. Garg H. Bhandari M. (2024). FDA-approved artificial intelligence and machine learning (AI/ML)-enabled medical devices: an updated landscape. Electronics. 13. doi: 10.3390/electronics13030498

[B39] Khan M. S. Hayat M. U. Khanam M. Saeed H. Owais M. Khalid M.. (2021). Role of biologically important imidazole moiety on the antimicrobial and anticancer activity of Fe(III) and Mn(II) complexes. J. Biomol Struct. Dyn 39, 4037–4050. doi: 10.1080/07391102.2020.1776156 32496965

[B40] Kim J. Ahn I. (2021). Infectious disease outbreak prediction using media articles with machine learning models. Sci. Rep. 11, 4413. doi: 10.1038/s41598-021-83926-2 33627706 PMC7904826

[B41] Kinsella R. J. Fitzpatrick D. A. Creevey C. J. Mcinerney J. O. (2003). Fatty acid biosynthesis in Mycobacterium tuberculosis: lateral gene transfer, adaptive evolution, and gene duplication. Proc. Natl. Acad. Sci. U.S.A. 100, 10320–10325. doi: 10.1073/pnas.1737230100 12917487 PMC193559

[B42] Kuang X. Wang F. Hernandez K. M. Zhang Z. Grossman R. L. (2022). Accurate and rapid prediction of tuberculosis drug resistance from genome sequence data using traditional machine learning algorithms and CNN. Sci. Rep. 12, 2427. doi: 10.1038/s41598-022-06449-4 35165358 PMC8844416

[B43] Kyrpides N. C. Eloe-Fadrosh E. A. Ivanova N. N. (2016). Microbiome data science: understanding our microbial planet. Trends Microbiol. 24, 425–427. doi: 10.1016/j.tim.2016.02.011 27197692

[B44] Lavalle S. M. Branicky M. Lindemann S. R. (2004). On the relationship between classical grid search and probabilistic roadmaps. Int. J. Robotics Res. 7, 59–75. doi: 10.1177/0278364904045481

[B45] Liu X. Kimmey J. M. Matarazzo L. De Bakker V. Van Maele L. Sirard J. C.. (2021). Exploration of bacterial bottlenecks and streptococcus pneumoniae pathogenesis by CRISPRi-seq. Cell Host Microbe 29, 107–120.e6. doi: 10.1016/j.chom.2020.10.001 33120116 PMC7855995

[B46] Luo H. Lin Y. Liu T. Lai F. L. Zhang C. T. Gao F.. (2021). DEG 15, an update of the Database of Essential Genes that includes built-in analysis tools. Nucleic Acids Res. 49, D677–D686. doi: 10.1093/nar/gkaa917 33095861 PMC7779065

[B47] Mathison B. A. Kohan J. L. Walker J. F. Smith R. B. Ardon O. Couturier M. R. (2020). Detection of intestinal protozoa in trichrome-stained stool specimens by use of a deep convolutional neural network. J. Clin. Microbiol. 58. doi: 10.1128/JCM.02053-19 PMC7269375 32295888

[B48] Milho C. Silva M. D. Alves D. Oliveira H. Sousa C. Pastrana L. M.. (2019). Escherichia coli and Salmonella Enteritidis dual-species biofilms: interspecies interactions and antibiofilm efficacy of phages. Sci. Rep. 9, 18183. doi: 10.1038/s41598-019-54847-y 31796870 PMC6890764

[B49] Mlynarczyk-Bonikowska B. Kowalewski C. Krolak-Ulinska A. Marusza W. (2022). Molecular mechanisms of drug resistance in staphylococcus aureus. Int. J. Mol. Sci. 23. doi: 10.3390/ijms23158088 PMC9332259 35897667

[B50] Mujeeb A. A. Khan N. A. Jamal F. Badre Alam K. F. Saeed H. Kazmi S.. (2020). Olax scandens mediated biogenic synthesis of ag-cu nanocomposites: potential against inhibition of drug-resistant microbes. Front. Chem. 8, 103. doi: 10.3389/fchem.2020.00103 32185160 PMC7058794

[B51] Munjal N. S. Sapra D. Parthasarathi K. T. S. Goyal A. Pandey A. Banerjee M.. (2022). Deciphering the interactions of SARS-coV-2 proteins with human ion channels using machine-learning-based methods. Pathogens 11, 259. doi: 10.3390/pathogens11020259 35215201 PMC8874499

[B52] Naidenov B. Lim A. Willyerd K. Torres N. J. Johnson W. L. Hwang H. J.. (2019). Pan-genomic and polymorphic driven prediction of antibiotic resistance in elizabethkingia. Front. Microbiol. 10, 1446. doi: 10.3389/fmicb.2019.01446 31333599 PMC6622151

[B53] Nemati M. Hamidi A. Maleki Dizaj S. Javaherzadeh V. Lotfipour F. (2016). An overview on novel microbial determination methods in pharmaceutical and food quality control. Adv. Pharm. Bull. 6, 301–308. doi: 10.15171/apb.2016.042 27766214 PMC5071793

[B54] Nguyen M. Brettin T. Long S. W. Musser J. M. Olsen R. J. Olson R.. (2018). Developing an in silico minimum inhibitory concentration panel test for Klebsiella pneumoniae. Sci. Rep. 8, 421. doi: 10.1038/s41598-017-18972-w 29323230 PMC5765115

[B55] Ochman H. Wilson A. C. (1987). Evolution in bacteria: evidence for a universal substitution rate in cellular genomes. J. Mol. Evol. 26, 74–86. doi: 10.1007/BF02111283 3125340

[B56] Olaya-Abril A. Jimenez-Munguia I. Gomez-Gascon L. Obando I. Rodriguez-Ortega M. J. (2013). Identification of potential new protein vaccine candidates through pan-surfomic analysis of pneumococcal clinical isolates from adults. PloS One 8, e70365. doi: 10.1371/journal.pone.0070365 23894641 PMC3720901

[B57] Omkar Khade K. S. (2024). The rhizosphere microbiome: A key modulator of plant health and their role in secondary metabolites production (Elsevier: Academic Press).

[B58] Pagès H. Gentleman P. A. R. Debroy S. Biostrings: Efficient manipulation of biological strings. Available online at: https://rdrr.io/bioc/Biostrings/.

[B59] Pajon C. Fortoul M. C. Diaz-Tang G. Marin Meneses E. Kalifa A. R. Sevy E.. (2023). Interactions between metabolism and growth can determine the co-existence of Staphylococcus aureus and Pseudomonas aeruginosa. eLife. 12. doi: 10.7554/eLife.83664.sa2 PMC10174691 37078696

[B60] Parthasarathi K. T. S. Munjal N. S. Dey G. Kumar A. Pandey A. Balakrishnan L.. (2021). A pathway map of signaling events triggered upon SARS-CoV infection. J. Cell Commun. Signal 15, 595–600. doi: 10.1007/s12079-021-00642-2 34487344 PMC8419830

[B61] Prokopenko D. Hecker J. Silverman E. K. Pagano M. Nothen M. M. Dina C.. (2016). Utilizing the Jaccard index to reveal population stratification in sequencing data: a simulation study and an application to the 1000 Genomes Project. Bioinformatics 32, 1366–1372. doi: 10.1093/bioinformatics/btv752 26722118 PMC5860507

[B62] Qu K. Guo F. Liu X. Lin Y. Zou Q. (2019). Application of machine learning in microbiology. Front. Microbiol. 10, 827. doi: 10.3389/fmicb.2019.00827 31057526 PMC6482238

[B63] Rajaraman S. Antani S. K. Poostchi M. Silamut K. Hossain M. A. Maude R. J.. (2018). Pre-trained convolutional neural networks as feature extractors toward improved malaria parasite detection in thin blood smear images. PeerJ 6, e4568. doi: 10.7717/peerj.4568 29682411 PMC5907772

[B64] Ren X. Palmer L. D. (2023). Acinetobacter metabolism in infection and antimicrobial resistance. Infect. Immun. 91, e0043322. doi: 10.1128/iai.00433-22 37191522 PMC10269061

[B65] Rousseeuw P. J. (1987). Silhouettes: A graphical aid to the interpretation and validation of cluster analysis. J. Comput. Appl. Mathematics 20, 53–65. doi: 10.1016/0377-0427(87)90125-7

[B66] Salim N. A. M. Wah Y. B. Reeves C. Smith M. Yaacob W. F. W. Mudin R. N.. (2021). Prediction of dengue outbreak in Selangor Malaysia using machine learning techniques. Sci. Rep. 11, 939. doi: 10.1038/s41598-020-79193-2 33441678 PMC7806812

[B67] Samuel G. Hogbin J. P. Wang L. Reeves P. R. (2004). Relationships of the Escherichia coli O157, O111, and O55 O-antigen gene clusters with those of Salmonella enterica and Citrobacter freundii, which express identical O antigens. J. Bacteriol 186, 6536–6543. doi: 10.1128/JB.186.19.6536-6543.2004 15375135 PMC516595

[B68] Schopf J. W. Kitajima K. Spicuzza M. J. Kudryavtsev A. B. Valley J. W. (2018). SIMS analyses of the oldest known assemblage of microfossils document their taxon-correlated carbon isotope compositions. Proc. Natl. Acad. Sci. U.S.A. 115, 53–58. doi: 10.1073/pnas.1718063115 29255053 PMC5776830

[B69] Sharp P. M. (1991). Determinants of DNA sequence divergence between Escherichia coli and Salmonella typhimurium: codon usage, map position, and concerted evolution. J. Mol. Evol. 33, 23–33. doi: 10.1007/BF02100192 1909371

[B70] Sudhakar P. Machiels K. Verstockt B. Korcsmaros T. Vermeire S. (2021). Computational biology and machine learning approaches to understand mechanistic microbiome-host interactions. Front. Microbiol. 12, 618856. doi: 10.3389/fmicb.2021.618856 34046017 PMC8148342

[B71] Tatusova T. Dicuccio M. Badretdin A. Chetvernin V. Nawrocki E. P. Zaslavsky L.. (2016). NCBI prokaryotic genome annotation pipeline. Nucleic Acids Res. 44, 6614–6624. doi: 10.1093/nar/gkw569 27342282 PMC5001611

[B72] Torrecilha R. B. Utsunomiya Y. T. Batista L. F. Bosco A. M. Nunes C. M. Ciarlini P. C.. (2017). Prediction of lymph node parasite load from clinical data in dogs with leishmaniasis: An application of radial basis artificial neural networks. Vet. Parasitol. 234, 13–18. doi: 10.1016/j.vetpar.2016.12.016 28115177

[B73] Tozzoli R. Caprioli A. Morabito S. (2005). Detection of toxB, a plasmid virulence gene of Escherichia coli O157, in enterohemorrhagic and enteropathogenic E. coli. J. Clin. Microbiol. 43, 4052–4056. doi: 10.1128/JCM.43.8.4052-4056.2005 16081950 PMC1233992

[B74] Umar M. F. Ahmad F. Saeed H. Usmani S. A. Owais M. Rafatullah M. (2020). Bio-mediated synthesis of reduced graphene oxide nanoparticles from chenopodium album: their antimicrobial and anticancer activities. Nanomaterials (Basel) 10, 1096. doi: 10.3390/nano10061096 32492878 PMC7353263

[B75] Valizadehaslani T. Zhao Z. Sokhansanj B. A. Rosen G. L. (2020). Amino acid k-mer feature extraction for quantitative antimicrobial resistance (AMR) prediction by machine learning and model interpretation for biological insights. Biol. (Basel) 9, 365. doi: 10.3390/biology9110365 PMC7694136 33126516

[B76] Varoquaux G. (2023). joblib.

[B77] Ventola C. L. (2015). The antibiotic resistance crisis: part 1: causes and threats. P T 40, 277–283. 25859123 PMC4378521

[B78] Verma R. Tiwari A. Kaur S. Varshney G. C. Raghava G. P. (2008). Identification of proteins secreted by malaria parasite into erythrocyte using SVM and PSSM profiles. BMC Bioinf. 9, 201. doi: 10.1186/1471-2105-9-201 PMC2358896 18416838

[B79] Vogan A. A. Higgs P. G. (2011). The advantages and disadvantages of horizontal gene transfer and the emergence of the first species. Biol. Direct 6, 1. doi: 10.1186/1745-6150-6-1 21199581 PMC3043529

[B80] Voter A. F. Callaghan M. M. Tippana R. Myong S. Dillard J. P. Keck J. L. (2020). Antigenic Variation in Neisseria gonorrhoeae Occurs Independently of RecQ-Mediated Unwinding of the pilE G Quadruplex. J. Bacteriol 202. doi: 10.1128/JB.00607-19 PMC6964745 31740492

[B81] Waskom M. L. (2021). seaborn: statistical data visualization. J. Open Source Software. 6. doi: 10.21105/joss.03021

[B82] Wu Y. Gadsden S. A. (2023). Machine learning algorithms in microbial classification: a comparative analysis. Front. Artif. Intell. 6, 1200994. doi: 10.3389/frai.2023.1200994 37928448 PMC10620803

[B83] Yee Y. C. Kisslinger B. Yu V. L. Jin D. J. (1996). A mechanism of rifamycin inhibition and resistance in Pseudomonas aeruginosa. J. Antimicrobial Chemotherapy 38, 133–137. doi: 10.1093/jac/38.1.133 8858465

[B84] Zarrineh P. Sánchez-Rodríguez A. Hosseinkhan N. Narimani Z. Marchal K. Masoudi-Nejad A. (2014). Genome-Scale Co-Expression Network Comparison across Escherichia coli and Salmonella enterica Serovar Typhimurium Reveals Significant Conservation at the Regulon Level of Local Regulators Despite Their Dissimilar Lifestyles. PloS One. doi: 10.1371/journal.pone.0102871 PMC4125155 25101984

