# Supplementary material for: A machine learning-based strategy to elucidate the identification of antibiotic resistance in bacteria
Source: Front Antibiot. 2024 Jun 18;3:1405296. doi: 10.3389/frabi.2024.1405296 (PMC11732175; doi:10.3389/frabi.2024.1405296)
Supplement: Supplementary file 1 [file DataSheet_1.zip › Supplementary Data.docx]

Supplementary Material

# Supplementary Data

Selection criteria for the pathogenic bacterial strains included in the study

In the present study the following criteria was used for the selection of pathogenic bacterial strains:

1. The complete list of pathogenic strains present in PATRIC database with host as human was downloaded
2. The list of strains was classified based on the antibiotic susceptible and resistant strains. The susceptible strains were excluded.
3. Thereafter, the antibiotic resistant strains with availability of complete assembled genomic sequences were extracted. For strains with more than one genome sequence available, the latest version updated on RefSeq as of July 2023 were selected
4. The genera with less than 20 antibiotic resistant strains available with complete assembled genomic sequences were excluded

Selection of optimum k-mer length

A multi fasta file with bacterial genomic sequences of 4 *Escherichia* strains, 3 *Acinetobacter* strains and 3 *Pseudomonas* strains was used to decide the optimum k-mer length. The bacterial genomes in the file were fragmented into k-mers of length 8, 10, 12 and 14. Subsequently each list of k-mers was individually filtered to remove duplicates. They were then mapped to the sequences and matrices containing the details on presence and absence of the k-mers in each strain was generated. The clustering was performed using Affinity propagation algorithm on the subset for each k-mer length matrix and the obtained results are as shown in Supplementary data table 1.

Supplementary data table 1: Output obtained on clustering the bacterial strains using matrices with different k-mer length as input

| k-mer length | Number of unique k-mers | Largest intermediate file size | Time to process intermediate file on 64GB RAM system | Number of clusters obtained | Silhouette coefficient | Calinski-Harabasz index | Davies-Bouldin index |
| --- | --- | --- | --- | --- | --- | --- | --- |
| 8 | 65536 | 124.3 megabytes | 4.0911 seconds | 1 | NA | NA | NA |
| 10 | 928915 | 221.5 megabytes | 22.5012 seconds | 3 | 0.77 | 149.43 | 0.21 |
| 12 | 4979855 | 782.2 megabytes | 1.9298 minutes | 3 | 0.93 | 348.62 | 0.08 |
| 14 | 10185216 | 1.7 gigabytes | 3.5667 minutes | 3 | 0.86 | 67.44 | 0.20 |

A k-mer length of 8 would be efficient in terms of time and resources required to process. However smaller k-mers would have a higher chance of occurrence in multiple sequences and hence it could not differentiate between the three genera. K-mer length of 10, 12 and 14 resulted in 3 clusters representing the strains from 3 different genera. In terms of time and system requirements, a k-mer of length 10 would be more efficient as compared to k-mer of length 12 and 14 as including the complete set of sequences would result in an exponential increase in the number of k-mers and the size of intermediate files.
